# “We Tie Up the Loose Ends”: Homecare Nursing in a Changing Health Care Landscape

**DOI:** 10.1177/2333393618816780

**Published:** 2018-12-14

**Authors:** Line Melby, Aud Obstfelder, Ragnhild Hellesø

**Affiliations:** 1 SINTEF, Trondheim, Norway; 2 Norwegian University of Science and Technology, Gjøvik, Norway; 3 University of Oslo, Oslo, Norway

**Keywords:** community nursing, organization and delivery of care, homecare, qualitative methodologies

## Abstract

During the last decades, the work of homecare nurses has been affected by several changes, including an aging population, the decentralization of health care, nursing recruitment crises and the scarcity of public resources. Few scholars have analyzed how these changes have impacted homecare nursing. In this article, we describe and discuss aspects of homecare nurses’ work, with specific focus on nurses “organising work.” We outline three phenomena that are increasingly occurring: (a) homecare nurses are frequently involved in negotiating care level and, consequently, what kind of care the patient will receive; (b) homecare nurses’ clinical practice has become increasingly advanced; and (c) and homecare nurses play an important role in coordinating care among interdependent actors. The article draws on material from participant observation and interviews with homecare nurses in two Norwegian studies. Changes in work practice increase the demand for nurses to be competent and have excellent organizational and collaborative skills.

## What Is Known About This Topic

- The practice of homecare nursing is changing due to demographic, health, and political developments.
- The concrete practice of homecare nursing is under researched, and we have little knowledge of the details of homecare nursing.

## What This Article Adds

- Homecare nursing practices take new forms in response to contextual changes, and new practices are becoming prominent.
- Homecare nurses’ practice is becoming increasingly advanced but is complicated by the lack of knowledge transfer from hospitals to homecare and to the challenges of working alone in the context of a private home.
- Homecare nurses are frequently involved in negotiating care level across and within municipalities and specialist health care and, consequently, what kind of care the patient will receive.
- Patient trajectories are more complex than before, and homecare nurses play a key role in coordinating care among interdependent actors.

## Introduction

During the last decades, the work of homecare nurses in Norway and other Western countries has undergone several changes, including changes in aging populations, changes in disease patterns, the decentralization of health care, nursing recruitment crises and the scarcity of public resources (Björnsdóttir, 2018; Gautun & Syse, 2017; Helse- og omsorgsdepartementet, 2015; Vabø, 2012). However, few scholars have analyzed what these changes means for homecare nurses’ work. Few studies on the performance of homecare nursing exist; therefore, the work of homecare nurses, specifically the less clinical parts of their work, has been largely invisible to outsiders (Allen, 2015; Latimer, 2000; Nardi & Engeström, 1999). This article aims to describe and discuss aspects of homecare nurses’ work, with a specific focus on nurses “organising work” (Allen, 2014). Based on empirical examples, we outline three responses of homecare nurses to changes in their work contexts, the specific organizational challenges they address and the skills that are increasingly needed to perform these activities. The article draws on material from participant observation and interviews with homecare nurses in two Norwegian studies conducted between 2011 and 2016.

### Literature Review

Regardless of where it is practiced, nursing is a multifaceted role in the sense that nurses must meet multiple professional demands and perform a wide range of tasks (Allen, 2015; Kim, 2010). summarizing the literature on nurses’ tasks, interaction with patients, “bedside care,” is most often described as the core of nursing work (Allen, 2004; Allen & Fabri, 2005; Mendes, da Cruz, & Angelo, 2015; Tønnesen & Nortvedt, 2012). Interacting with patients involves assessing the patient’s situation, which includes diagnosing illnesses and planning and implementing proper interventions. Moreover, nurses must apply strategies and practices to improve care outcomes, such as implementing evidence-based practices, fulfill a professional commitment (the duty of care), and have an ethnical awareness of nursing practices (Mendes et al., 2015).

Caring for the patient also includes conducting what Allen (2014, 2015) calls “organizing work,” which essentially entails planning and executing the individual patients’ entire care trajectory. Organising work involves collaborating and coordinating with providers on different organizational levels and units and communicating and collaborating with family carers (Hvalvik & Dale, 2013), who in particular play an important role for home-dwelling patients. Organizing work also entails carrying out information management tasks, such as record keeping and information sharing, as well as “care space governance,” which includes maintaining a focus on quality, safety, and cost effectiveness (Mendes et al., 2015).

The general features of nursing work are relevant as a background for our analysis of homecare nursing. However, homecare nursing has specific features that make it particularly challenging. First, homecare nursing takes place within the patient’s home; thus, the home becomes both a workspace and a private, domestic space, which sets particular conditions under which care is provided (England & Dyck, 2011). Homecare nurses must adapt to patients’ various values and lifestyles and establish trust and provide care in highly individualized contexts (Hvalvik & Dale, 2013; Spiers, 2002). Providing care in the patient’s home affects social interaction and requires a balancing effort between providing care and maintaining the patient’s autonomy, self-determination, and dignity (cf. Holmberg, Valmari, & Lundgren, 2012). Providing care in the patient’s home also creates a sense of intimacy, and nurses invest in emotional labor and act with sentiment despite a lack of financial compensation (Johnson, 2015). Second, homecare nurses mostly work alone. Although being autonomous and independent may allow homecare nurses to effectively provide individualized care to patients, homecare nurses work in isolation (Spiers, 2002) and have little access to professional support. Third, homecare nurses work with increasingly advanced cases (Björnsdóttir, 2014), which means that homecare nurses frequently need to contact other health care workers both for obtaining professional advice and for coordinating services. In particular, the discharge process from hospital to home has proven to be challenging, as it leaves homecare nurses with an insufficient understanding of the patient’s situation (Grönroos & Perälä, 2005; Hvalvik & Dale, 2013; Olsen, Østnor, Enmarker, & Hellzén, 2013).

In the research literature, the therapeutic relationship with patients, whether it is exercising bedside care for hospital-bound patients or providing support at point of care for home-dwelling patients, is frequently portrayed as the core act in exercising nursing. Different types of organizing work are often depicted as inferior to direct patient contact, taking time away from care (Allen, 2014, 2015; Banerjee, Armstrong, Daly, Armstrong, & Braedley, 2015). Björnsdóttir (2018), however, describes these organizing efforts as a prerequisite for nurses’ provision of safe and high-quality health care. In her study of homecare, she found that nurses spent much time creating and maintaining a net of assistance and support around the patients to help them to continue living in their own home. To build such nets around the patients requires competencies, skills, and formal systems for connecting organizations, providers, and family carers. Direct interaction with patients and organizing their care may be seen as two sides of the same coin, and both are necessary for providing good nursing.

To explore the enactment of homecare nursing and the intertwinement of homecare practice and contextual changes, we draw on insights from Translation Mobilization Theory (TMT). TMT (Allen & May, 2017) explains the mechanisms through social actions are mobilized, the relationship between these practices, and the institutional contexts in which they are accomplished (p. 4). TMT draws on insights from ethnographic work on the social organization of health care work (Allen, 1997, 2004, 2009) and on Normalization Process Theory (NPT; May, 2013; May & Finch, 2009), which aims to explain how implementation takes place and how practices become integrated into a wider sociotechnical context.

TMT has three core components: (a) *Project*: a sociotechnical ensemble of institutionally sanctioned strategic activity mobilized across a distributed action field. In our case, this means the activity of homecare nursing. (b) *Strategic action field*: the institutional context in which projects emerge and are progressed and which provide the normative and relational frame for collective action. Such a field further entails organizing logics, structures, materials/technologies, and interpretative repertoires. The strategic field in our case is the Norwegian health care system, health policies, the staffing of homecare, and the tools and technologies that homecare nurses use in their work. (c) *Mechanisms of mobilization and institutionalization*: processes through which agents operating within a strategic action field mobilize projects, drive action, and perform institutions. These processes are of particular relevance in our analysis of how homecare nurses respond to changes in their work context. Such processes include the following:

- Object formation: practices that fabricate and configure the objects of knowledge and practice and enroll them into an actor network.
- Articulation work: practices that assemble and align diverse actors (people, knowledge, materials, etc.) and through which object trajectories are mobilized (cf. Strauss, Fagerhaug, Suczek, & Wiener, 1997).
- Translation: practices that enable objects to be shared and differing viewpoints, local contingencies, and multiple interests to be accommodated to enable concerted action.
- Reflexive monitoring: practices through which actors evaluate a field of action to generate situational awareness of project trajectories.
- Sensemaking: practices through which actors order, construct, and mobilize projects and enact institutions (Allen & May, 2017 p. 8).

The concepts are useful for understanding and articulating how homecare nurses make sense of their work and what strategies they apply when the context in which they work is constantly changing. The concepts link actors and actions with the overall structure of health care, emphasizing the relationship between practices and the institutional context, which is highly relevant in our analysis of homecare nursing in a changing health care landscape.

### Setting: the Strategic Field of Norwegian Homecare

Community health care service in Norway is governed and financed by the municipalities, and municipalities are obliged by law to ensure that its inhabitants receive “necessary health and care services” that are well coordinated and safe (Helse- og omsorgsdepartementet, 2011). Municipal care is divided into institutional care, such as nursing homes, and health care provided in the patient’s home, such as homecare nursing. Overall, the main objective of homecare nursing is to ensure that home-dwelling patients’ fundamental needs are met. An increasing number of Norwegian inhabitants are receiving homecare services, and the number is expected to increase (Mørk, Sundby, Otnes, & Wahlgren, 2014). The patients vary in terms of age. Circa 60% of them are above pension age (67 years). The largest patient group is between 80 and 89 years (31% of the patient population). Patients who are older than 81 to 89 years are likely to receive institutionalized care. Longitudinal statistics also indicate that the number of younger patients (which refers to patients who are below pension age) is increasing (Mørk et al., 2014). Many of these patients have chronic illness and are multimorbid, and some of these patients are physically disabled and consequently need advanced clinical care, including a multitude of medications and/or other care measures. In 2012, the Norwegian government implemented a health care reform (known as the Coordination Reform), in which responsibilities were transferred from the specialist health care service (hospitals) to the municipalities (Helse- og omsorgsdepartementet, 2009). The reform encouraged early discharge from hospital; as a consequence, homecare patients are sicker than before (Schou, Helgesen, & Hofstad, 2014). Moreover, due to their complex conditions, homecare patients need contact with a range of other health care providers, such as general practitioners (GPs), out-patient clinics, rehabilitation institutions, and physiotherapists, in addition to homecare nurses.

The developments in Norwegian homecare are not unique to the Norwegian context, but represent general trends in Western health care. Thus, our study’s findings should have relevance for understanding homecare practices in other countries.

## Method

### Study Design

This article is based on data collected for two of our previous research studies. In Study 1, Bridging the Information Gap in Patient Transition (BIG; 2010–2014), we studied the interaction between homecare nurses, GPs, and hospital staff, as well as how homecare nurses used an electronic messaging system as a collaborative tool. Study 2, Interaction via ICT (SIKT) (2013–2016), was a follow-up to the first study, but focused more specifically on the use of collaboration to treat patients in need of both homecare and hospital treatment.

### Methods and Setting Study 1

Study 1 was conducted in three municipalities in Norway, which varied in size. We combined observations of homecare nursing work and semi-structured interviews, and data collection took place between May 2011 and January 2012. Access to and permission for observing and interviewing were granted by the head of the homecare services in the municipalities. After permission was obtained, a contact person approached and informed eligible nurses who met the inclusion criteria about the study. Nurses were included in the study if they had been in their position for more than 6 months; this ensured that the participating nurses had some experience. The contact person also organized the observations and interviews.

The observations covered 97 home visits, which resulted in a total of 85 hr of observation. The first author and the third author conducted the observations. By providing rich data on events in context, observations uncover the enactment of a practice or a project (Tjora, 2012). The observations were mostly organized in the same way across the three municipalities. We started the day with a morning meeting during which we observed all of the staff together, updated each other on the patients, coordinated work, and delegated tasks before heading out to the patients’ homes. Together with each assigned nurse, we visited patients in their homes. During the visits, we asked for clarifications of what took place or discussed situations that occurred with the nurses we followed. Sometimes the patients or next of kin were involved in the discussions. After the home visits, we returned with the nurses to the homecare office. Each day ended with conducting an interview with the nurse we had observed and sometimes with other homecare nurses. Occasionally we would also observe nurses involved in office work; normally, one experienced nurse would use the day to coordinate the patients’ appointments with other professionals, update medication lists, and order new medications. To record the observations, we took field notes. Our goal was to produce field notes that described the situations as accurately as possible.

The interviews included 43 persons and were conducted by the first author, the third author, and a PhD student. There were 23 homecare nurses, 11 general practitioners, five medical secretaries, and four project managers for the e-communication implementation. The interviews with homecare nurses would typically begin with an open-ended question: tell us about a typical day at your work. Concrete themes addressed how the nurses assessed the patients’ needs, if they had experienced changes in their work over time—and, if so, what kind of changes—and how collaboration was experienced. All but one interview were conducted by two interviewers; one of the researchers led the interview and ensured that all themes in the interview guide were covered. The other researcher took notes and asked clarifying questions.

### Methods and Setting Study 2

In Study 2, we only conducted interviews, which took place in 2014. The setting was three municipalities (one of which was the same as in Study 1) and one hospital. Recruitment of interviewees followed the same procedure as in Study 1. In total, 41 persons were interviewed. These included 24 interviewees in homecare (the rest worked at the hospital): 12 worked as homecare nurses, whereas 12 worked as case handlers or other administrative staff. The interviews were conducted by the first author, the third author, and a researcher. As in Study 1, interviews were carried out by two researchers together.

Most municipalities today use a purchaser-provider-based system, which means that the case handlers in a municipality “buy” services from the providers (e.g., homecare) in the same municipality. The case handlers are thus the ones who formally decide what kind of municipal health and social care the inhabitants receive; consequently, case handlers have much knowledge of the needs of the patients and the services offered by the municipality. The purchaser-provider model requires good collaboration between purchasers and providers to continuously adapt services to the patients’ needs.

### Analysis

The analytical themes presented in the results section illustrate the interplay between the overall development in the health care system and the practical accomplishment of homecare nursing. The findings presented in this article reflect only parts of the data collected in the studies, and other publications highlight other aspects of homecare in detail (see, Melby, Brattheim, & Hellesø, 2015; Melby & Hellesø, 2014). We have selected data that allowed us to uncover and analyze *stories of change* to present in-depth insight regarding homecare nursing practices. Both in formal interviews and in conversations that occurred during the observations, nurses shared their experiences of the changed nature of homecare with us. The concept of “change” was therefore a guiding concept when going through the data material. After conducting discussions among the authors, three overall themes were identified. When presenting the themes, we highlight typical situations for each of the themes and give examples of caring/practice situations based on the field notes as well as excerpts from the interviews. Interviewees have been given pseudonyms.

### Ethics

Both projects were reported to the Norwegian Centre for Research Data (Projects 26,230/2011 and 37399/2014), and they were approved by the management in the municipalities. All the homecare nurses gave their written informed consent. Because observations were made in the patients’ home, all patients were asked in advance to approve that a researcher would follow the nurse into the patients’ home. All patients signed an agreement that granted us permission to observe the work of nurses in their homes.

## Results

In this section, we present the study’s results, which are organized in three themes. Each theme represents activities that homecare nurses increasingly carry out:

- Homecare nurses are frequently involved in negotiating care level and, consequently, what kind of care the patient will receive. This theme shows how nurses try to understand the patient’s needs, communicate those needs and match patients’ needs to available services.
- Homecare nurses’ practice has become increasingly advanced, and homecare nurses now perform procedures that previously took place only in hospitals. This theme points to the importance of systems for knowledge transfer from hospitals to homecare and to the challenges of working alone in the context of a private home.
- Patient trajectories are more complex than before, and homecare nurses play an important role in coordinating care between interdependent actors. This theme depicts the major role homecare nurses play in being mediators between actors who lack good systems for information transfer and communication.

### Negotiating Care Level and Services

A main finding from our studies was that homecare nurses take active parts in negotiations regarding the patients’ service level, both through intra-municipal negotiations and in communication with the hospitals concerning the transfer of patients from hospital to home. Vignette 1 shows an example of homecare nurse Tina’s first visit to a patient recently discharged from a rehabilitation stay and how she assesses the patient’s care needs:

***Vignette 1*:**

After lunch, we go to Mrs. Jackson, a woman in her eighties who lives with her husband in an elegant two-storey house. Mrs. Jackson came home from a four-week rehabilitation stay in a nursing home only ten minutes ago. Tina is only slightly oriented about how the stay has been and the status of Mrs. Jackson’s health situation. I get the impression that the visit is mainly for establishing how Mrs. Jackson is feeling, what kind of help from community care she has been granted, and what she considers her needs to be. We enter the house and are shown into the living room. Mrs. Jackson suffers from mild dementia, but seems to be in a good shape otherwise. She is in a very good mood. Her husband is cognitively sharp, but has severe back problems and can only walk very slowly. Mr. Jackson and the nurse have a long conversation about the needs of Mrs. Jackson. Mrs Jackson sits at the table, but does not participate in the conversation. One problem is that Mr. Jackson finds it difficult to take his wife to the GP when that is needed, and he wants the GP to come to their home. They agree that Tina will ask the GP if he can come and see Mrs. Jackson at home. They also agree upon an increase in the number of homecare visits. From having visits twice a day, Mrs. Jackson will now have visits four times a day. We leave the house and aim for the GP’s office to ask if he can see Mrs. Jackson at home. (Observation B3)

In the example, Tina and Mrs. Jackson’s husband discuss and agree upon the number of visits per day needed for Mrs. Jackson. The nurse must also address the couple’s difficulties of going to the GP. The nurse’s work can be seen as a practice of “object formation,” the configuration of “the case” of Mrs. Jackson (Allen & May, 2017). By investigating both the needs of Mrs. Jackson’s and her husband, the nurse tries to determine what services Mrs. Jackson needs, how often she needs help, what other actors need to be enrolled in the patient’s care network, and how to connect the various actors.

Our studies also revealed that homecare nurses play a significant role in the shaping of trajectories by informing caseworkers (purchasers) about the patients’ situation, which is another input for object formation. This happened on a regular basis through a function in the electronic patient record system, through which providers can notify case handlers about changes in patients’ needs. It also happened when patients were about to be discharged and case handlers needed more information about a patient. Our studies indicate that homecare nurses are more influential in discussions with the caseworkers than with hospital staff. The negotiations regarding transfer from hospital to home can be particularly challenging. In these conversations, the hospital’s need for discharge as soon as the patient is done with specialist treatment meets community care’s need for preparations, including getting the necessary equipment in place in the patient’s home (e.g., a hospital bed). It is important to note that legally it is the hospital’s responsibility to decide when the patient is ready for discharge. However, for patients in need of homecare services, the service quality can be substantially lower if homecare nurses have not been able to prepare the homecoming. Therefore, discussions regarding patients’ needs and an agreement regarding the time of discharge is therefore necessary (Hellesø & Melby, 2013).

Frequently, negotiations were nevertheless not an option. One homecare nurse said the following: “We had a patient that we requested to stay in the hospital a little bit longer. And they [the hospital] insisted that he should be discharged. So, we think there is more room for negotiation in the other departments than in the XX department.” We do not know the hospital’s motivation for insisting on discharge, but Norwegian hospitals are increasingly experiencing productivity pressure, so they aim to discharge patients early. Interestingly, we also heard examples of the opposite: homecare arranged a service in the patient’s home, but the hospital judged it as insufficient and refused to discharge the patient:

It seems like there is most tumults with patients with psychiatric diagnoses when it comes to discharges. And if there is one time where we were close to having a quarrel with the hospital, it was related to a psychiatric patient that we thought could be discharged to home—because the patient was cared for by one [nurse] during the day and one [nurse] during the evening. And community care nursing during the night. The hospital did not think it was justifiable. But I am unsure whether we managed to explain how much services the patient actually had at home. But they refused to discharge him on that Friday, and we were sitting here, fully staffed. (Nurse A, municipality B)

However, interviewees also told several stories of more peaceful negotiations over services, in which the hospital would postpone discharge so that homecare nurses could properly arrange for the patient to come home. One nurse said, “We do some negotiations and reach an agreement. The hospital will then discharge the patient the following day instead of today.”

### Performing Increasingly Advanced Clinical Practices

Another main finding in our studies—and perhaps the most visible one—was that homecare nurses care for much more ill patients than they did earlier and consequently must perform more advanced clinical assessments and procedures. This can be related to the public efforts of keeping patients out of hospitals and providing care in the municipality. Because tasks that traditionally were performed by specialists in hospitals are now carried out by homecare nurses, the significance of knowledge transfer from hospitals to the municipalities has increased. Another complicating factor in performing advanced clinical practices is that homecare nursing always takes place in the patient’s home and, consequently, work must be conducted within a setting which is not originally designed for exercising care. Vignette 2 describes a situation from one of the observations, in which a nurse must perform a procedure she has not performed before:

***Vignette 2*:**

The forth visit this day is to Mrs. Hughes, a widow in her seventies. She was discharged from hospital the day before, and this is the first visit from homecare after she came home. Mrs. Hughes lives alone and is dependent on homecare to assist her with various tasks. The nurse, Carrie, is informed that Mrs. Hughes has had her gallbladder removed and her bile duct cut. Apart from that, she knows little about the patient’s current condition and needs. When Carrie talks to Mrs. Jackson, it becomes clear that the patient has a drain tube to remove excess fluid from her wound. She informs Carrie that she needs to empty the drain and measure the amount of fluid in it. The patient explains in detail what the hospital physician told her needs to be done with the drain, but Carrie has not been directly informed by the hospital and seems quite puzzled. It is obvious that Carrie has never seen a drain like this before and is unsure of how to perform the task. Regardless, they go into the bathroom to do the job. (Observation A3)

The vignette represents a not uncommon example in which a nurse enters a patient’s home and must perform relatively advanced tasks for which she is not prepared. As the vignette indicates, situations like this typically occur because of a lack of information from the hospital to homecare nursing about how to follow up on the patient’s needs and how to carry out a specific procedure.

The displacement of patients from hospital to municipal care leads to a need for increased competences of various kinds among staff. Such a change necessitates transfer of competence between hospitals and homecare. In other words, procedures traditionally conducted in hospitals must be translated and made doable in private homes. However, knowledge transfer and translation are not always conducted systematically, as reported in several interviews:

Nurse B: “The communication concerning procedures for wound care is poor. Often, we do not have a procedure at all. Then we call [the hospital nurses] and complain a bit. If they cannot help us, we contact the physician who is responsible for prescribing the procedure.”

Nurse C: “We do have quite some experience ourselves, because there are many wounds. But it is not our responsibility to make the procedure. If the patient gets an infection, we are not responsible, because it is the [hospital] doctor’s job to make the procedure.” (Municipality C)

The data showed several examples of poor education of homecare staff and a lack of communication between hospital and homecare when preparing for discharge of patients from hospital to home.

### Coordinating Care Between Interdependent Actors

The third overall finding from our studies was that homecare nurses coordinate patients’ services and that the frequency of performing this job increases as the complexity of health care increases. Currently, there is no dedicated professional role for maintaining oversight and managing and coordinating services along the caring trajectory. Throughout data collection, we witnessed numerous examples of homecare nurses having to take on the coordinator role to compensate for the lack of coordinative mechanisms in the health care system. Vignette 3 shows a typical example:

***Vignette 3*:**

The second visit this morning goes together with nurse Ellie to Mr. Andrews, a widower in his seventies. Homecare has taken over the responsibility for his medications. Mr. Andrews’ blood sugar level is at the moment very unstable, and Ellie’s job is to measure his blood sugar level and make sure he takes his medications, which are administered through a multi-dose drug dispensing system. Ellie is also going to cook him breakfast. Ellie and Mr. Andrews start to talk about his medications. He complains about side effects from Glucophage, a medication for his diabetes. Because he experienced nausea after taking the medication, his GP ended the medication. Now it turns out that Mr. Andrews is back on Glucophage, and he is unsure of how this has happened. He thinks perhaps it is homecare staff who have told the GP that he must start up with the medication again. Ellie is unaware of the whole thing, so she looks through the nursing documentation from the previous days, which are stored in the patient record system on her PDA. After scrolling back some days, she informs Mr. Andrews that it his GP that has reinstated Glucopage. She tells Mr. Andrews that she will call the GP afterwards and talk to him about Mr. Andrews’ medications. We leave the house, and Ellie documents in the PDA in Mr. Andrews patient record. Ellie will go back to Mr. Andrews at 1 PM to give him a B12 shot, and she hopes she can inform him then. (Observation A1)

In particular, it is difficult to coordinate patients’ medications across multiple health providers (e.g., hospitals, GPs, and community care; Holly & Poletick, 2014; Lyngstad, Melby, Grimsmo, & Hellesø, 2013). In Vignette 3, only community care and the GP are involved, but practices can be even more complex when the hospital is involved as well. We found that the most critical situations concerning medications happened when patients were discharged from the hospital to home, and there were inconsistencies between the medication lists in the hospital, the GP, and homecare. The GP is responsible for home-dwelling patients’ medications, but most often GPs are informed about hospitalization later than homecare, and thus in practice it becomes the homecare nurses’ job to clear up inconsistencies so that the patient will receive the right medications:

We compare [the new list from hospital] with our medication list from before they were admitted to hospital [. . .] If we see that it seems utterly wrong, or the hospital has not been aware that the patient was on certain medications, we contact the GP. If everything looks fine, we will make the changes and send a copy to the GP, informing him/her about the changes. (Nurse D, municipality C)

Another example described how patients were regularly discharged from the hospital without the necessary medications and how the pharmacy was closed at the time homecare came to see them. In these cases, which were described as very frustrating, nurses had to borrow medications from other patients (or from a nearby nursing home) and contact the GP for a prescription on the following day.

In general, homecare nurses described much of their activity as to “tie up the loose ends and collect information.” One nurse said that because she works so closely with patients—in the patients’ home—she felt that homecare nurses have “essential information” about patients.

The examples show that homecare nurses act as coordinators for ensuring that home-dwelling patients receive their medications and act as intermediaries between GPs, hospitals, and patients. Research has shown that nurses often orchestrate and manage the work of others, specifically doctors’ work (Allen, 2004), and this is visible in our studies as well. Transitions between hospitals and the home challenge the ideal of coherent and continuous trajectories and cause a great need for coordination. However, our studies also showed that coordination between actors *within* the municipality is demanding. Both sorts of coordination are articulated and mobilized as prominent practices in homecare nursing.

## Discussion

Our studies have highlighted three areas of homecare work that are increasingly performed. The themes were derived from “stories of change” in our data and show how “organising work” has gained a central place in homecare nursing. We argue that changes in work practice are responses to contextual changes in the overall health care system. Seen in relation to TMT, changes in the strategic action field lead homecare staff to apply various strategies and resources and mobilize new activities to cope with these changes (cf. Allen & May, 2017). With the advent of new activities, there is also a need for increased competencies and new systems and routines for supporting nurses in their tasks. The processes that drive actions as outlined in TMT, will consequently increase in intensity in times of rapid changes. For example, will the need for articulation work for aligning actors and mobilizing care trajectories increase and become more challenging when the institutional context is changing. Likewise, will sensemaking—literally practicses of making sense of what is going on in day to day work—become more demanding when, for example, new forms of division of labor are initiated, making practice more complex. In the article, we have shown examples of such practices and strategies, while trying to relate them to the changes taking place in homecare.

Research on nursing practices has shown that nurses are active participants in shaping and managing patients’ trajectories (Allen, 2015; Latimer, 2000; Spiers, 2002; Strauss et al., 1997). In our studies, we found that homecare nurses negotiate with other providers as well as informal carers regarding the service level (e.g., home or hospital) and the amount of services required in the home. The negotiations take place both through their own initiative and the initiative of others. Our results indicate that homecare nurses can greatly influence the patient’s service when at home, but have less influence on the time of discharge even though some hospital departments have good dialogue with homecare services and allow for joint decision making.

The influence of nurses on home-dwelling patients’ services found in this study is in line with that found in Vabø’s (2012) studies. She concluded that even though the purchaser-provider model was introduced to secure a separation between those contracting services and those providing the services, in practice purchasers and care staff collaborate to manage the fluctuating needs and everyday dilemmas of care. Our studies support this finding. We found that homecare nurses regularly adjusted and updated decisions via the electronic patient record system based on their knowledge of the patients’ needs. Today, when home-dwelling patients are sicker and have more complex conditions than before, homecare nurses’ advice on the patients’ service needs are increasingly important. The negotiation over service level can be seen as an act of object formation (Allen & May, 2017), in which nurses use their knowledge of the patient and enroll them into an actor network that can provide the most appropriate service level. Our studies showed that negotiations and clarifications of patients’ conditions between homecare and hospital staff happened frequently yet were not always sufficient for accommodating the home or providing homecare nurses with sufficient knowledge so patients could be properly cared for. When patients are transferred from the hospital to home, hospital staff need to know that the patients have adequate care services at home, and homecare nurses need information about the patient’s status to accommodate care. We found great variety between cases and informants in terms of how well the communication between hospital and homecare functioned. Previous studies have underlined that this interface is a challenging area (Grönroos & Perälä, 2005; Hellesø & Fagermoen, 2010). In our study, we found that electronic communication tools, like e-messaging, may ease communication and patient care planning (see Melby et al., 2015). The increase in rapid discharges from the hospital increases the chance that patients experience more readmissions (Gautun & Syse, 2017), which makes care trajectories discontinuous and fragmented. In turn, this means that the transfer and communication between the hospital staff and homecare nurses must happen more frequently and that homecare nurses’ role in the transfer process becomes even more important. Research has shown that homecare nurses may experience the transfer process as demanding and that there is room for improvement in the collaboration across hospitals and municipalities (Hvalvik & Dale, 2013), which is also supported by our findings.

Nurses increasingly take on advanced clinical work that was previously performed in hospitals. This development is encouraged by the health authorities, and represents a form of task shifting that has occurred in other countries (Denton, Brookman, Zeytinoglu, Plenderleith, & Barken, 2015). The question is how the development is accommodated in terms of educating homecare staff and knowledge transfer from hospitals to municipalities. Studies indicate that there is a discrepancy between the advanced competence expected in policy documents and the actual competence nurses possess (Bing-Jonsson, Foss, & Bjørk, 2016; Gautun & Syse, 2017). Our studies add knowledge to this gap. We found that competence varied among nurses, and there were several occasions in which nurses called for more training and information about a specific procedure. In particular, the nurses in our studies called for better communication and knowledge transfer from specialist health care to community care. The latter is obliged by law as a duty of the specialist health care system (Helse- og omsorgsdepartementet, 1999). Bing-Jonsson et al. (2016) concluded their study by stating that there is a general lack of opportunities for competence development in the health and care sector. Because nurses must perform increasingly advanced tasks, it is worrisome if nurses are not able to increase their competence, which, in turn, challenges nurses’ professionalism. The challenges of performing advanced care work is reinforced by the context in which homecare is performed. A private home is not arranged for performing advanced care, which can make it difficult to prepare sterile areas and have enough space to perform procedures (cf. Corbin & Strauss, 1985; England & Dyck, 2011). In addition, homecare staff mostly work alone, so they cannot discuss practices or procedures while trying to perform them, as exemplified in Vignette 2.

The interviews demonstrated how nurses continuously evaluated the information they received from their collaborating partners (e.g., the hospital) or the lack of such. This happened regularly due to the large number of patients being rapidly discharged from hospital. Contextual changes and a lack of good routines for information and knowledge transfer therefore made it necessary for nurses to develop a continuous awareness of patients’ needs as well as if, and how, homecare should respond to those needs (Allen & May, 2017; May, 2013).

Our studies showed that homecare nurses spend a lot of time and effort on coordination work. Scholars have argued that hospital nurses are “conductors of care” through the acts of coordination and delegation (cf. Latimer, 2000). Nurses in homecare hold this role, and the coordinator role is increasingly important as patient’s trajectories become more complex (e.g., by more frequent readmissions to the hospital). The original illness trajectory concept, as developed by Strauss et al. (1997), was developed in a hospital context. Later, the concept was expanded to better address the situation of patients/persons living at home and patients with more fluctuating trajectories and/or longer trajectories, like the patients in our studies. These developments seek to address that the original trajectory concept excludes extra complexities and unpredictabilities that emerge when patients receive services from many providers during a complex course of illness (Allen, Griffiths, & Lyne, 2004; Gallagher et al., 2013; Hannigan & Allen, 2013). Allen et al. (2004) introduce the concept “caring trajectory” to reflect the combined health and social care contributions made in hospitals and in community care, as well as by informal carers, while Gallagher et al. (2013) coin the term “problematic trajectories.” These trajectories are characterized by the presence of, for example, multimorbidity and complex care regimes. Our studies have shown that there are no routine trajectories for patients receiving homecare; rather, trajectories are problematic and require much effort to manage.

Homecare nurses coordinate care both with other health care and social care professionals and informal carers, as well as the patient. Negotiations of services, as described in the first result theme, should also be seen as a part of the coordinator job. By doing so, the nurses take on a significant role in articulating care trajectories. They align actors and actions to progress the trajectory. The nurses in our studies emphasized the problem that patients were discharged from the hospital to home without having medications—or prescriptions—with them. This is a typical situation that requires a lot of coordination work from homecare nurses, which can include telephoning the hospital, updating the patient’s multidose drug dispensing list, electronically informing the GP about the changes and asking him or her to update and prescribe new medications, going to the pharmacy and fetching medications, explaining the new medication regime to the patient, and so on. Ensuring that the patient gets the right medications thus requires coordinating and aligning actors’ perceptions of a situation (what is the right medications for this patient?) as well as engaging in concrete patient–nurse encounters. In daily work, the three homecare nursing practices described in this article often appear simultaneously (and must be understood together), as all three practices are necessary to deliver good patient care.

## Strengths and Limitations

The strength of this study is that we have analyzed data from two studies conducted at different points of time, which allowed us to obtain a comprehensive understanding of the challenges faced by homecare nurses. It also provided us with rich data that allowed us to analyze the changes in the health care sector. Despite its strengths, the study has several limitations. One limitation is that data originally were not collected with the purpose of exploring how nurses responded to contextual changes. If that had been our main research question, we may have obtained even more detailed data on changes in work practices. Another limitation is that we conducted a limited number of observations, and by expanding the number of observations, we may have gained better insight into the practical accomplishment of current homecare nursing. However, we have discussed findings with other researchers and with homecare nurses and their leaders to validate our findings. It should be noted that qualitative data usually is not suited for generalizations. However, in this study we have data from different homecare districts representing different sizes and regions in Norway. Our findings are similar across these settings, which indicates that the trends we identify in the article are relevant for other homecare districts as well.

## Conclusions and Implications for Practice

Changes in the health care system imply a shift in health care professionals’ work. In this article, we provide examples of how nurses cope with and react to the changes in the system, and we have shown three types of work practice that homecare nurses are spending more time conducting. Another Norwegian study on homecare nursing concludes that the nurses’ ambitions for excellent care are threatened by the context that stems from developments in the health care system, thus leading to the lack of cooperation, competence, and continuity (Hvalvik & Dale, 2013). Our studies raise some of the same concerns, but we also find that homecare nurses are skilled and flexible in the exercise of care and are adaptive to change. In particular, we learned that organizing work is increasingly conducted. Such work is a remedy for fragmented patient trajectories and for facilitating collaboration among the patients’ carers.

In this article, we have tried to show that clinical work at point of care and organizing work must be seen as intertwined, and in order to provide safe care for the patient, the care services must be thoroughly organized. Nurses play a significant role in organizing patients’ trajectories. This has implications for nursing education and for nursing practice development. More competence in organizing and collaboration is needed both in municipalities and hospitals, and it should be appreciated that such practices are crucial in the provision of high-quality nursing.
